# A patient with Owren disease requires pancreatic surgery: A case report

**DOI:** 10.1097/MD.0000000000036562

**Published:** 2023-12-15

**Authors:** Yang Jun, Qiu Ming, Luo Nai-Wen, Cao Lei, Fan Yu-Dong, Wang Shu-Guang, Wang Yao

**Affiliations:** a Department of Abdominal Surgery, Guiqian International Hospital, Guiyang City, China; b Department of Precise Medical Center, Guiqian International Hospital, Guiyang City, China.

**Keywords:** bleeding risk, coagulation dysfunction, congenital factor V deficiency, intraoperative bleeding, pancreatic surgery

## Abstract

**Rationale:**

Coagulation factor V deficiency is rare, and perioperative management of patients with this condition is particularly important, especially during major abdominal surgery. We present a case of a patient with pancreatic duct stones combined with coagulation factor V deficiency. We share our perioperative management experience.

**Patient concerns:**

A 31-year-old man presented with recurrent upper abdominal pain for 2 years.

**Diagnoses:**

The diagnosis of pancreatic duct stones in the patient has been established through abdominal computed tomography and magnetic resonance imaging examinations. The diagnosis of factor V deficiency was initially identified through coagulation function tests, revealing significant prolongation of both aPTT and PT. Subsequent testing of coagulation factors and inhibitors demonstrated that the patient has a deficiency in coagulation factor V. Finally, genetic testing revealed that the factor V deficiency in this case is hereditary.

**Interventions:**

The patient underwent a partial resection of the pancreatic head, and FFP was infused 1 hour before surgery. 600 mL of FFP was instilled 1 hour before the start of surgery along with 10 U of cryoprecipitate. and 600 ml of FFP were added during surgery. Postoperative treatment included intermittent FFP supplemental infusion in the first 5 days after surgery while monitoring the coagulation function.

**Outcomes:**

The patient underwent a successful surgery without any abnormal bleeding or oozing during the procedure. The postoperative recovery was smooth, with no abnormal bleeding.

**Lessons:**

Patients with a deficiency of coagulation factor V are not contraindicated for surgery. Appropriate Fresh Frozen Plasma (FFP) replacement therapy can ensure the safe conduct of the surgical procedure. For patients with abnormal blood coagulation function, we recommend testing for coagulation factors and inhibitors, as well as performing genetic testing for abnormal coagulation factors, which can provide guidance on marriage and childbirth.

## 1. Introduction

Abnormal blood coagulation function is often contraindicated in many surgical procedures, especially high-risk surgeries such as pancreatic surgery, and the human coagulation pathway is divided into endogenous and exogenous coagulation pathways. Abnormalities in any pathway or a common pathway can lead to coagulation abnormalities. Coagulation factor V deficiency is a rare condition. As an important factor in the coagulation pathway, its deficiency can lead to prolonged activated partial thromboplastin time (aPTT) and/or prothrombin time (PT). How can the perioperative period be managed reasonably to reduce the risk of bleeding?

## 2. Case presentation

**Figure 1.**
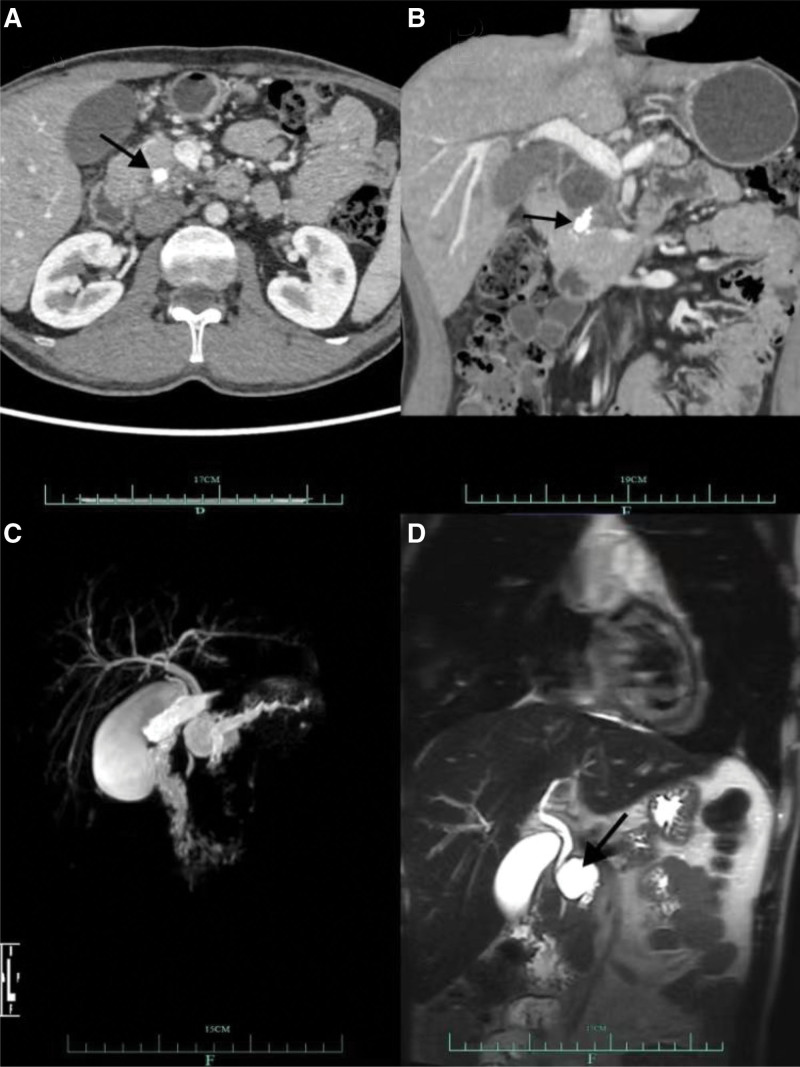
(A) High-density intraductal calculus in the pancreatic head region observed on computed tomography. (B) Pancreatic duct stones and cysts located above the head of the pancreas are visible in the coronal plane. (C) Magnetic resonance cholangiopancreatography revealed significant thickening of the pancreatic duct and cysts in the head region of the pancreas. (D) Dilatation of the pancreatic duct and cysts in the head of the pancreas.

A 31-year-old male patient sought medical attention at our hospital for recurrent upper abdominal pain for 2 years. In the past 2 years, the diagnosis of chronic pancreatitis combined with pancreatic duct stones has been confirmed in many hospitals, and abnormal coagulation dysfunction has been found. After standardized treatment for pancreatitis, pain can be alleviated; however, it still frequently recurs. There was no significant history of bleeding or family history of bleeding. During physical examination, the patient weighed 57.4 kg, and there were no abnormal signs except mild epigastric tenderness; after admission, the patient had mild epigastric tenderness, no rebound tenderness, or muscle tension. Abdominal computed tomography and magnetic resonance cholangiopancreatography (Fig. 1) revealed chronic pancreatitis with pancreatic duct stones. Coagulation laboratory tests showed a PT of 22.2 seconds (↑), international normalized ratio (INR) of 1.96 (↑), aPTT of 68.4 seconds (↑), fibrinogen of 4.06 g/L (↑), and antithrombin (AT)-III (141% ↑). Other tests, such as blood cell count, liver and kidney function tests, antineutrophil cytoplasmic antibodies, antinuclear antibody spectrum, anticardiolipin antibody, and thromboelastogram (TEG), identified no abnormalities.

## 3. Analysis and management during the perioperative period

### 3.1. The cause of coagulation abnormalities

The patient had no history of bleeding, gum bleeding, melena, or hematochezia after minimal trauma, and no family history of coagulation abnormalities. However, multiple aPTT and PT examinations demonstrated obvious prolongation of these parameters, excluding laboratory errors. Therefore, this might be due to either a decrease in or lack of coagulation factors or the presence of coagulation factor inhibitors. After examination at our hospital, we found that the patient’s coagulation factor V (FV) was significantly reduced (Table 1) and there were no coagulation factor inhibitors in the blood. The antineutrophil cytoplasmic antibodies, antinuclear antibody spectrum, and anticardiolipin antibody test results were negative. We found that coagulation factor V, which plays a role in the common coagulation pathway, was reduced, resulting in prolonged aPTT and PT.

**Table 1:** Changes in coagulation factor V after infusion of cold precipitate and fresh frozen plasma.

	Infusion of cryoprecipitate 10 units	Infusion of fresh frozen plasma 10 units
	V factor	V factor
Before infusion	1.9	0.7
1h after infusion	5.1	20.1
24h after infusion	3.3	6

### 3.2. Evaluating the risk of bleeding

The patient underwent multiple reexaminations for coagulation function and TEG, as shown in the figure below. To evaluate changes in coagulation factors, aPTT, PT, and TEG after the transfusion of cold precipitate and fresh frozen plasma (FFP), reexaminations were performed 1 and 24 hours after transfusion (Table 1).

Correcting coagulation function could be achieved through transfusion of FFP or cold precipitate, and the patient’s TEG was normal; thus, the risk of bleeding during surgery could be controlled.

### 3.3. Hereditary versus acquired disorder

**Figure 2.**
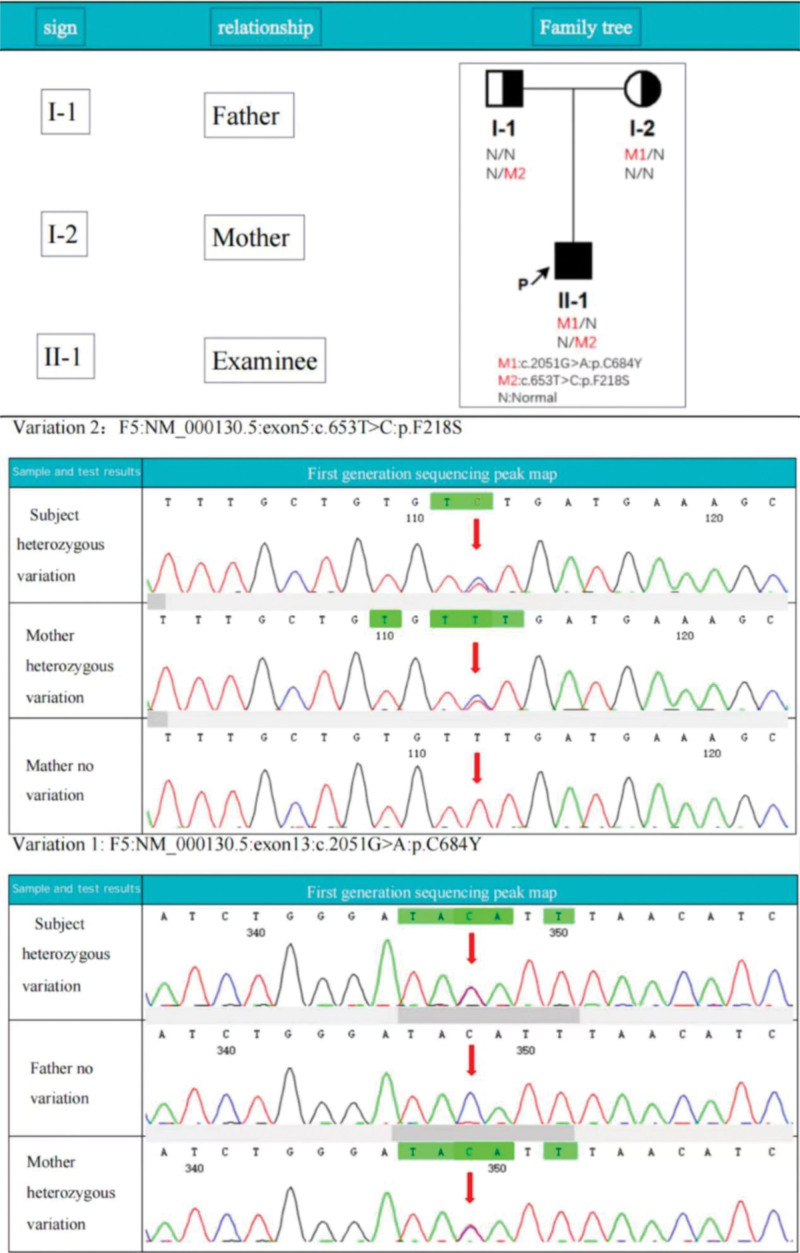
Genetic variation pedigree graph and variation validation graph.

Genetic testing was performed on the patient and his parents (Fig. 2). According to the test results, 2 heterozygous variants were detected in *F5*: variant 1 (c.2051G>A:p. C684Y, in which the 2051st position of the mutation was changed from base G to base A, resulting in the 684th codon-coding tyrosine instead of cysteine) and variant 2 (c.653T>C:p. F218S, which changes T to C at position 653 of the cDNA, results in a change in the codon at position 218 from encoding serine instead of phenylalanine). Variant 1 was inherited from the subject’s mother and variant 2 was inherited from the subject’s father, forming a compound heterozygous variant.

### 3.4. Preoperative and intraoperative management

**Figure 3.**
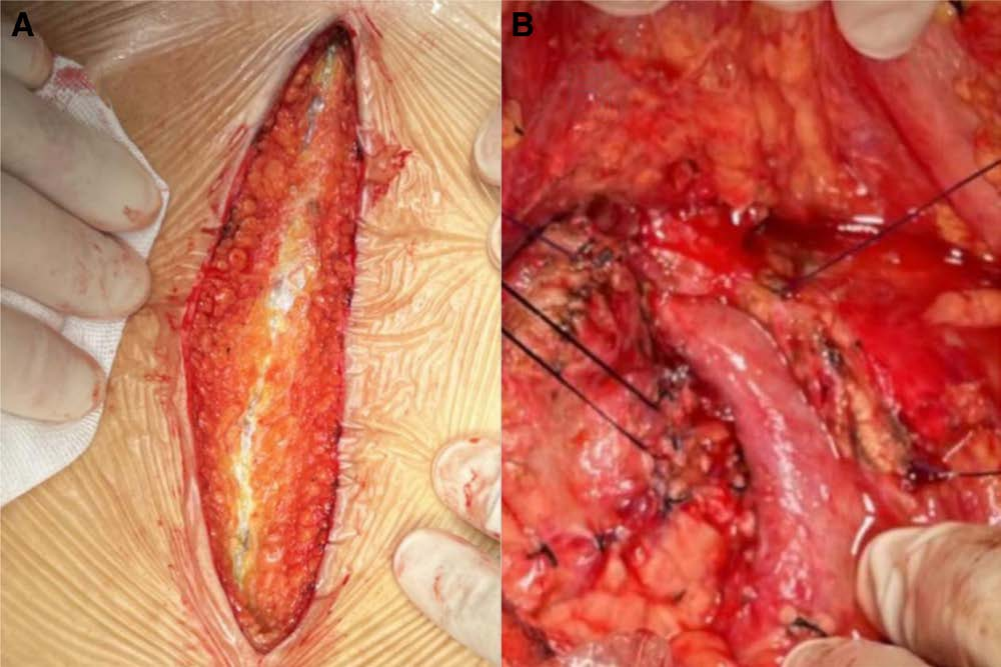
(A) There were no abnormal bleedings from the abdominal incision. (B) There were no signs of abnormal bleeding in the operative field after partial resection of the pancreatic head.

We infused FFP and cryoprecipitate for exploratory purposes and evaluated the effects of these 2 blood products on coagulation (Tables 2a and 2b). We observed that the aPTT and PT improved significantly 1 hour after infusion and recovered to their initial levels after 24 hours. We continued to test the patient’s TEG to further assess the risk of bleeding, and found that the patient’s TEG was normal. According to relevant research, the dose for replacement therapy using FFP is generally 15 to 25 mL/kg(2). Meanwhile, to avoid overloading the volume of liquid infusion, based on the previous results of our diagnostic experiments (see Table 1), we empirically used a portion of the cold precipitate instead of fresh frozen plasma. There are no reports in the literature on the use of cold precipitates as substitute therapy. Intraoperatively, 600 mL of FFP was instilled 1 hour before the start of surgery and 10 U of cryoprecipitate and 600 ml of FFP were added during surgery. Surgery was performed without complications or abnormal intraoperative bleeding. The intraoperative TEG measurements were normal. The intraoperative images are shown in Figure 3.

Postoperative treatment included intermittent FFP supplemental infusion in the first 5 days after surgery while monitoring coagulation function. There was no bleeding from the postoperative incision, abdominal drainage, or a decrease in the hemoglobin level. The patient recovered and was discharged from the hospital after the surgery. There were no postoperative bleeding events, and the patient recovered well until the current telephone follow-up was 12 months.

## 4. Discussion

Factor V deficiency (FVD) is a rare coagulation disorder divided into hereditary coagulation factor V deficiency (HFVD) and acquired factor V deficiency. The incidence of heterozygotes and homozygotes in the general population is approximately 1 in 1000 and 1 in 1000,000 individuals, respectively. Human FV is a high-molecular-weight (approximately 330 × 10^3^) single-chain glycoprotein encoded by the *F5* gene, synthesized primarily in the liver. FV deficiency results in significantly prolonged aPTT and PT.^[1]^ Generally, FV activity is approximately half of the normal level in heterozygotes, who are usually asymptomatic. In contrast, homozygotes show certain clinical symptoms; however, their heterogeneity is very high. Common symptoms include soft tissue or mucosal hemorrhages, fatal hemorrhages, profuse (nasal and menstrual) hemorrhages, bleeding during major and minor surgical and dental procedures, hematomas, and gastrointestinal, pulmonary, and intracranial hemorrhages.^[2]^

Hereditary FVD caused by defects in the *F5* gene is the most common FV-related bleeding disorder. More than 160 *F5* gene mutations have been recorded, most of which are missense or nonsense.^[3]^ Coagulopathy is often a contraindication for many surgeries, especially major surgeries. It is particularly important to assess the risk of bleeding and to determine whether these patients can benefit from surgery.

Coagulation factor V (FV), an important coagulation cofactor, is synthesized in hepatocytes and megakaryocytes. FV is mainly distributed in the plasma(80%) but is also present in platelets (20%).^[4]^

This patient needed surgical treatment due to chronic pancreatitis and pancreatic duct stones, but multiple laboratory tests revealed that PT and aPTT were significantly prolonged. Further examination revealed that the patient’s FV was significantly reduced, which was confirmed to be HFVD by genetic testing, aPTT, PT correction tests, and FV inhibitor detection in both the patient and his parents. The patient was a heterozygous carrier, who was usually asymptomatic but also suffered from surgical diseases, such as chronic pancreatitis with pancreatic duct stones, requiring surgical treatment. The risk of intraoperative and postoperative bleeding was abnormally high, and the surgery was performed cautiously. Our center has reviewed and summarized the relevant literature on HFVD. Although the PT and partial thromboplastin time are prolonged, they can be corrected using FFP. Surgical operations are unavoidable in certain cases; thus, preoperative, intraoperative, and postoperative intermittent plasma replacement therapy is required to correct coagulation abnormalities. However, no consensus has been reached regarding the risk assessment of surgical bleeding with abnormal coagulation function. Research has shown that very low levels of FV (<1%) can produce a large amount of thrombin, thus significantly improving hemostasis.^[5,6]^ Consequently, it is generally believed that the level of FV activity required for normal hemostasis can reach 25%. Furthermore, Duckers et al found that FV activity of 10% can produce normal amounts of thrombin.^[6]^

From a pathophysiological point of view, vascular injury and coagulation dysfunction are the most common causes of bleeding, and intraoperative and postoperative bleeding caused by coagulation dysfunction are not uncommon. As the pancreas has an abundant blood supply, in pancreatic inflammatory diseases, the quality of blood vessels decreases, microdamage becomes difficult to avoid, and the risk of intraoperative and postoperative bleeding greatly increases. Therefore, we targeted and individualized FFP transfusion based on the patient’s PT, aPTT, and FV activity monitoring before, during, and after surgery to avoid bleeding-related complications. Through a retrospective analysis of this case, we verified the important role of FFP transfusion and cryoprecipitate in coagulation dysfunction in this patient. Treatment of FV deficiency is currently based on transfusions of fresh frozen plasma or the use of coagulation factor concentrates, such as Octaplas®.^[7,8]^ 200 mL of FFP only contains 0.7 to 1.0 IU/mL for all coagulation factors. Additionally, cryoprecipitate mainly supplements coagulation factors VIII, vWF, fibrinogen, and factor XIII. The level of coagulation factor V was also low. Therefore, surgery requires the transfusion of a higher volume of FFP or cryoprecipitate to achieve the desired FV level, which can easily cause hypervolemia and cardiac overload.

Another concern in perioperative management is the timing of alternative therapy because FV has a half-life of 12 to 36 hours. Most patients receive intermittent treatment only before invasive surgery, in which the dose is usually 10 to 20 mL/kg of FFP and is recommended to achieve a target safe activity level of 15% to 20% prior to the invasive procedure.^[9,10]^ Platelet transfusion may be necessary in severe cases.^[11]^ Infusion for 5 to 10 days and early or late alternative therapy may result in waste of medical resources or poor treatment results. Moreover, under physiological conditions, FV has dual pro-coagulation and anticoagulation effects. Hence, in addition to judging the bleeding risk according to PT and aPTT, such patients are at risk of thrombosis.^[12]^ Furthermore, in cases of high-risk postoperative thrombosis, venous thrombotic events should be considered in patients with a high risk of postoperative thrombosis. However, replacement treatment options for cryoprecipitation have not been reported and there is value for further research.

Through this study, the diagnosis of Factor V deficiency is not difficult, as the collection of the patient’s medical history can reveal clues in cases of abnormal blood clotting function. As a common factor in both the intrinsic and extrinsic pathways of blood clotting, factor V deficiency often manifests as a significant prolongation of aPTT and PT, with most patients showing no obvious bleeding tendency. This study reveals that it is necessary to examine both clotting factors and clotting factor inhibitors in patients with abnormal blood clotting function. Factor V deficiency can be classified as acquired or inherited, and can be clearly identified through genetic testing, which can also reveal its inheritance pattern. Currently, there is no cure for congenital factor V deficiency, and the main treatment is replacement therapy. Perioperative management mainly involves replacement therapy with Factor V: for invasive surgery, intermittent treatment is administered, with an initial dose of 15 to 25 mL/kg, followed by 15 to 25 mL/d/kg for 5 to 10 days, which can safely be used for major invasive surgeries.

## 5. Conclusion

Patients with FVD have a high risk of bleeding during surgery and reasonable perioperative management can reduce the risk of bleeding and even perform complex surgical procedures. Preoperative evaluation of coagulation function and substitution of blood products is important to ensure surgical safety. For patients with abnormal blood coagulation function, we recommend testing for coagulation factors and inhibitors, as well as performing genetic testing for abnormal coagulation factors, which can provide guidance on marriage and childbirth. However, this case report has limitations and is not universally applicable. Its guidance for clinical practice is limited, but it still holds some reference value. Further research on the safety of surgical procedures for patients with coagulation factor V deficiency is still worth investigating in the future.

**Table 2a:** Changes in coagulation function indicators after 10 units cold precipitation infusion.

	Before infusion	1 h after infusion	24 h after infusion	48 h after infusion
PT	22.4	20.1	21.4	22.3
INR	1.99	1.76	1.91	2.01
aPTT	63.2	54.5	54.5	61.4

aPTT = activated partial thromboplastin time, INR = international normalized ratio, PT = prothrombin time.

**Table 2b:** Changes in various coagulation function indicators after infusion of 1200 mL fresh frozen plasma.

	Before infusion	1 h after infusion	24 h after infusion	48 h after infusion
PT	22.6	15.7	19.4	20.8
INR	2.05	1.27	1.67	1.83
aPTT	59.1	43.4	53.6	54.4

aPTT = activated partial thromboplastin time, INR = international normalized ratio, PT = prothrombin time.

## Acknowledgments

Special thanks to the Precision Medicine Center for their contribution to the genetic testing of the patients and Professor Wang Shu-guang for his guidance in writing this article.

## Author contributions

**Project administration:** Wang Su-Guang.

**Supervision:** Fan Yu-Dong, Wang Su-Guang, Wang Yao.

**Writing – original draft:** Yang Jun.

**Writing – review & editing:** Qiu Ming, Luo Nai-Wen, Cao Lei.
